# Changes in atherosclerotic plaque composition assessed by MRI in the superficial femoral artery with two years of lipid lowering therapy

**DOI:** 10.1186/1532-429X-13-S1-P382

**Published:** 2011-02-02

**Authors:** Amy M West, AJ Pesch, Neesurg Mehta, Justin D Anderson, Frederick H Epstein, Craig H Meyer, Klaus D Hagspiel, Stuart S Berr, Nancy L Harthun, Jennifer R Hunter, Joseph M DiMaria, John M Christopher, Christopher M Kramer

**Affiliations:** 1 University of Virginia, Charlottesville, VA, USA

## Objective

We hypothesized that changes in atherosclerotic plaque composition with lipid lowering therapy could be measured in patients with peripheral arterial disease (PAD) using MRI of the superficial femoral artery (SFA).

## Background

MRI of atherosclerotic plaque components in the carotid arteries correlates with histology, is reproducible, and can monitor lipid lowering therapy.

## Methods

43 patients with PAD (age 63±9, ABI 0.72±0.14) were imaged at baseline and annually for 2 years after beginning lipid lowering therapy. Twenty statin-naïve patients were randomized to simvastatin (S, n=11) or simvastatin+ezetimibe (S+E, n=9). Patients already on statins not at LDL cholesterol goal had open-label ezetimibe added (E, n=23). MRI of a single slice of the SFA was performed on Siemens Avanto 1.5T scanner with a proton-density weighted image (TR 1100ms, TE 7.6ms), T1-W image (TR 700ms, TE 7.7ms) and T2-W image (TR 2000ms, TE 85ms), using slice thickness 3mm, matrix 256x256 pixels, FOV 150mm, flip angle 180°. Image location was repeated over time using the distance of the imaged slice from the bifurcation of the common femoral artery. Image quality scale from 1-4 was used to grade the images, excluding 6 studies for score ≤ 2 in more than one image. VesselMASS software was used to measure plaque components (lipids, calcium, loose matrix and fibrous tissue) based on published critieria for appearance on T1/T2/PDW images. Changes in parameters were assessed with ANOVA repeated measures model and correlation with two-tailed Pearson.

## Results

LDL at baseline in the statin-naïve patients (S and S+E) was 127±34mg/dl and fell at one year (82±37, p<0.001) and was unchanged at two years (84±44). There was no difference in LDL between S and S+E. The LDL in E at baseline was 93±21mg/dl, decreased significantly at one year (77±35, p<0.001), and was similar at year 2 (74±27). Total cholesterol changes paralleled that of LDL. See Table 1 for changes in wall area and plaque components. The amount of plaque lipids correlated with wall area (r=0.44, p<0.003) for for all studies. Intra-observer variability examined in 10 patients with an intra-class correlation coefficient of 0.99 for wall area and 0.88 for plaque components.

**Table 1:** Changes in plaque over time

	Baseline	Year 1	Year 2
**Wall Area (cm^2^)**			
S and S+E	0.33±0.14	0.32±0.14	0.32±0.14
E	0.31±0.05	0.29±0.12	0.29±0.12
**Lipids (cm^2^)**			
S and S+E	0.05±0.04	0.04±0.04	0.03±0.03*
E	0.04±0.05	0.04±0.05	0.02±0.03*
**Calcium (cm^2^)**			
S and S+E	0.004±0.007	0.005±0.010	0.009±0.016
E	0.011±0.026	0.011±0.022	0.014±0.023
**Loose Matrix(cm^2^)**			
S and S+E	0.05±0.07	0.04±0.05	0.03±0.03
E	0.03±0.06	0.04±0.07	0.04±0.07
**Fibrous** Tissue **(cm^2^)**			
S and S+E	0.23±0.13	0.23±0.12	0.25±0.13
E	0.23±0.09	0.20±0.10	0.22±0.10

*ANOVA p<0.05

## Conclusion

LDL lowering therapy over 2 years decreases plaque lipids without a significant change in plaque area or other components as demonstrated by MRI.

**Figure 1:**
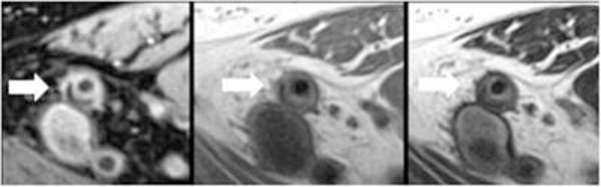
Representative PDW (left), T1-W (center) and T2-W (right) images of the SFA (arrow) in a study patient

